# Self-disorders in schizophrenia spectrum disorders

**DOI:** 10.1192/j.eurpsy.2025.2059

**Published:** 2025-08-26

**Authors:** I. M. M. Soerensen, J. Nordgaard, I.-M. Moelstroem, R. Handest, A. R. Rasmussen, K. E. Sandsten, J. Thalbitzer

**Affiliations:** 1 Psychiatry Region Zealand, University of Copenhagen, Roskilde; 2 University of Copenhagen, Copenhagen, Denmark

## Abstract

**Introduction:**

The term *self-disorder* investigated in this study refers to a disorder of the minimal self. It is a disturbance at the basic pre-reflective level typical in schizophrenia spectrum disorder, These disturbances are often present in the prodromal face of schizophrenia.

In 2003 Sass and Parnas proposed the ipseity-disturbance model (*ipseity also sometimes termed minimal self, basic self, experimental self, and for-meness).* Since 2003 Parnas and colleagues have published EASE (Examination of anomalous Self-experience): a semi-structured interview guide to allow for a systematic, qualitative and quantitative assessment of self-disorders.

The EASE-scale consist of a checklist of 57 items divided into 5 domains: 1) Cognition and Stream of Consciousness, 2) Self-Awareness and Presence, 3) Bodily Experiences, 4) Demarcation/Transitivism and 5) Existential Reorientation. The domains serve to structure the interview and to aid the interviewer to navigate and cover all 57 items during the interview.

In the last 20 years, several studies using EASE have supported self-disorders to be a central psychopathological feature of schizophrenia. A systematic review from 2021 showed that self-disorders hyper- aggregate in schizophrenia spectrum disorders but not in other mental disorders. However, self-disorder aggregation and distribution in between schizophrenia spectrum disorders have not been investigated before.

**Objectives:**

The purpose of this study is to compare overall EASE-sums in types of schizophrenia spectrum disorders including subtypes of schizophrenia (ICD10).

We hypothesize that there will not be a significant difference in overall EASE-score, reflecting self-disorder to be a core disturbance in schizophrenia spectrum disorders.

Furthermore, we plan to perform explorative statistical analysis on item-level of the EASE-scale, to investigate whether self-disorders presents differently and characteristically in-between schizophrenia spectrum disorders.

**Methods:**

Data is pooled from 6 different studies, totaling 236 patients.

All patients were examined using the EASE-guide by a professional clinicians who were trained in the semi-structed EASE-interview. The patients were furthermore assessed thoroughly for psychopathology and diagnosed according to ICD10 and DSM-IV or V.

**Results:**

Ongoing

**Conclusions:**

Ongoing

**Disclosure of Interest:**

None Declared

